# Real-time monitoring of secretory protein traffic for investigating trafficking regulators of ACE2 in living cells

**DOI:** 10.1016/j.jbc.2025.110593

**Published:** 2025-08-12

**Authors:** Chang Zhang, Mengxin Yang, Yanzun An, Muyi Liu, Huilong Li, Ruzhou Zhao, Xiaopan Yang, Linfei Huang, Yanhong Zhang, Renjun Peng, Hui Zhong, Xinlong Yan, Congwen Wei, Luming Wan

**Affiliations:** 1 Laboratory of Advanced Biotechnology, Beijing Institute of Biotechnology, Beijing, China; 2 Institute of Physical Science and Information Technology, Anhui University, Hefei, China; 3 Beijing Key Laboratory of Environmental and Viral Oncology, College of Chemistry and Life Science, Beijing University of Technology, Beijing, China; 4 Honours College of Capital Normal University, Capital Normal University, Beijing, China; 5 Department of Nuclear Radiation Injury and Monitoring, The PLA Rocket Force Characteristic Medical Center, Beijing, China; 6 Beijing Youngen Technology Co, Ltd, Beijing, China

**Keywords:** intracellular trafficking, cell surface receptor, cholesterol, analytical chemistry, adaptor protein, ACE2, NPC1, HiBiT tag

## Abstract

The cellular secretory pathway is a fundamental physiological process; yet a precise, efficient, and quantitative method for monitoring this process remains seldom explored. In this study, we achieved real-time monitoring of the secretion of angiotensin-converting enzyme 2 and programmed death-ligand 1 by integrating the HiBiT tag with the classic Retention Using Selective Hooks system. Through this strategy, we identified and validated Niemann–Pick complex 1 as a lysosomal adaptor involved in the angiotensin-converting enzyme 2 trafficking process. We propose that the strategy offers a valuable tool for the precise and rapid identification of diverse trafficking regulators and therapeutic compounds targeting various receptors or cytokines, thereby advancing the field of cellular transport research.

The cellular secretory pathway is a fundamental physiological process that refers to the transport of newly synthesized proteins to their target compartments. In eukaryotes, conventional protein secretion is primarily mediated by secretory vesicles, which transport cargo through the endoplasmic reticulum (ER) and the Golgi apparatus to the plasma membrane (PM) or extracellular space. This process is essential because it directs proteins, such as cytokines, chemokines, hormones, and receptors, to their sites of action, where they perform crucial physiological and pathological functions (1).

As the receptor of severe acute respiratory syndrome coronavirus 2, angiotensin-converting enzyme 2 (ACE2) is widely expressed across various human tissues and cell types, and its expression levels are critical for viral entry (2, 3, 4, 5). However, the mechanisms underlying its secretion remain poorly understood, and no targeted strategies have been developed to modulate this process, primarily because of the inherent complexity of studying secretion dynamics (6, 7, 8).

Several classic methods have been developed to investigate secretory trafficking, with the RUSH (Retention Using Selective Hooks) system being the most advanced technique (9, 10, 11, 12). The RUSH system exploits the high-affinity interaction between streptavidin (Str) and a streptavidin-binding peptide (SBP) to reversibly retain an enhanced GFP (EGFP)–tagged reporter within the ER. Subsequent addition of biotin, which competitively displaces SBP from Str, triggers reporter release. This controlled release enables quantification of secretion kinetics and status by monitoring EGFP localization dynamics *via* fluorescence microscopy (10).

Researchers have conducted impressive studies using RUSH system, driving significant process in the field of cellular trafficking (13, 14, 15). However, the classic RUSH assay requires the acquisition and quantification of numerous fluorescence images, which poses challenges for achieving precision and efficiency, particularly constraining its use in high-throughput screening applications. Boncompain *et al.* (16) utilized the classic RUSH system to screen and validate three molecules from over 4000 compounds that inhibit HIV infection by targeting CC chemokine receptor 5 secretion. To our knowledge, this is the only reported study to apply the RUSH technique to high-throughput screening.

HiBiT is an 11-amino-acid peptide tag developed by Promega in recent years. When HiBiT-tagged proteins on the cell surface or in the extracellular space bind to the exogenously added Large BiT, a NanoBiT luciferase is generated, enabling the detection and quantification of HiBiT-tagged proteins in living cells (17, 18). Building on this advantage, we propose substituting the EGFP in the RUSH reporter with HiBiT, thereby converting the fluorescent signal into a quantifiable luciferase signal, which would facilitate real-time, quantitative monitoring of the reporter trafficking.

In this study, we combined the HiBiT technology with the classic RUSH system to investigate the secretion process of ACE2 as a model. Using a plate reader, we achieved real-time monitoring of the secretion dynamics, enabling the precise and efficient identification of the trafficking regulators. We identified Niemann–Pick complex 1 (NPC1) as a key adaptor mediating the lysosomal degradation of ACE2, thereby elucidating the molecular mechanism by which cholesterol upregulates ACE2. Notably, this strategy was also applied to monitor the secretion process of programmed death-ligand 1 (PDL1), a key immune checkpoint on tumor cell surface, highlighting the significant potential of this approach in advancing the field of cellular trafficking research.

## Results

### Rationale and construction of HiBiT-integrated RUSH plasmids adapted for ACE2 and PDL1

**Figure 1:**
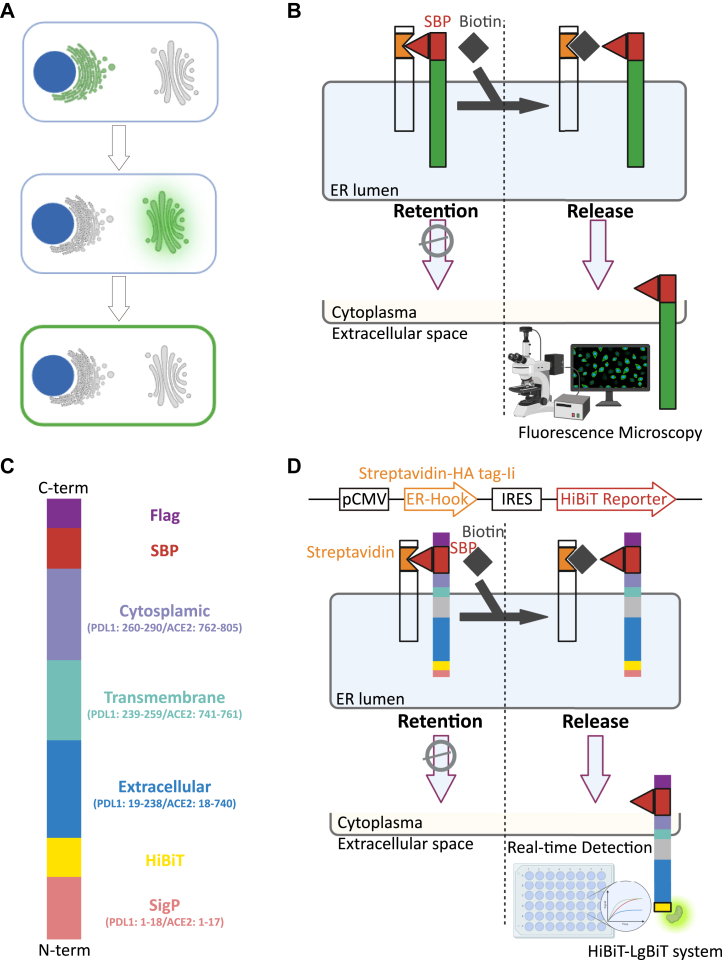
**Rationale and construction of this study.***A*, conventional RUSH system utilizes fluorescent proteins to monitor the secretion and trafficking of target proteins. *B*, biotin competes with streptavidin-binding peptide (SBP) for binding to streptavidin (Str), triggering the release of the EGFP-tagged reporter. *C*, design of reporters for ACE2 and PDL1. *D*, The ER-localized hook and HiBiT reporter were engineered into a single plasmid using an internal ribosome entry site (IRES) element to construct HiBiT-integrated RUSH plasmid, enabling real-time monitoring of protein secretion in living cells with a plate reader for detection. ACE2, angiotensin-converting enzyme 2; EGFP, enhanced GFP; ER, endoplasmic reticulum; PDL1, programmed death-ligand 1; RUSH, Retention Using Selective Hooks.

The classic RUSH assay relies on EGFP to visualize the secretion of proteins from the ER to PM or extracellular space (Fig. 1*A*). This method utilizes the interaction between Str and SBP to hook an EGFP-tagged reporter to the ER. Upon the addition of biotin, which competes for Str binding, the reporter is released, enabling the determination of secretion speed and status through EGFP localization *via* fluorescence microscopy (Fig. 1*B*).

For the design of HiBiT-integrated RUSH plasmid, the membrane topology of reporter protein should be carefully considered to achieve successful anchoring to the hook while also ensuring that the HiBiT tag is ultimately exposed to the extracellular space. To be more specific, the Str-Ii_SBP-EGFP-Golgin84 plasmid (addgene #65303) was selected as the basis for designing HiBiT-integrated RUSH plasmids targeting ACE2 and PDL1. This plasmid backbone employs an ER-localized hook system comprising a fusion of human invariant chain (Ii, for ER localization) with Str (for binding reporter), further tagged with HA for immunoblotting detection. For design of reporters, the SBP module was positioned in the cytoplasmic region to ensure its interaction with the ER-located Str–Ii hook. For these two type I transmembrane proteins (7, 19), the HiBiT tag was incorporated at the N terminus and positioned directly downstream of the signal peptide to prevent its cocleavage during signal peptide processing. In addition, a FLAG tag was added to the C terminus of constructs for immunoblotting or immunoprecipitation (IP) analysis (Fig. 1, *C* and *D*). The two plasmids were designated as Str–Ii_HiBiT-ACE2-SBP-FLAG and Str–Ii_HiBiT-PDL1-SBP-FLAG, respectively.

### Validation of HiBiT-integrated RUSH strategy

**Figure 2:**
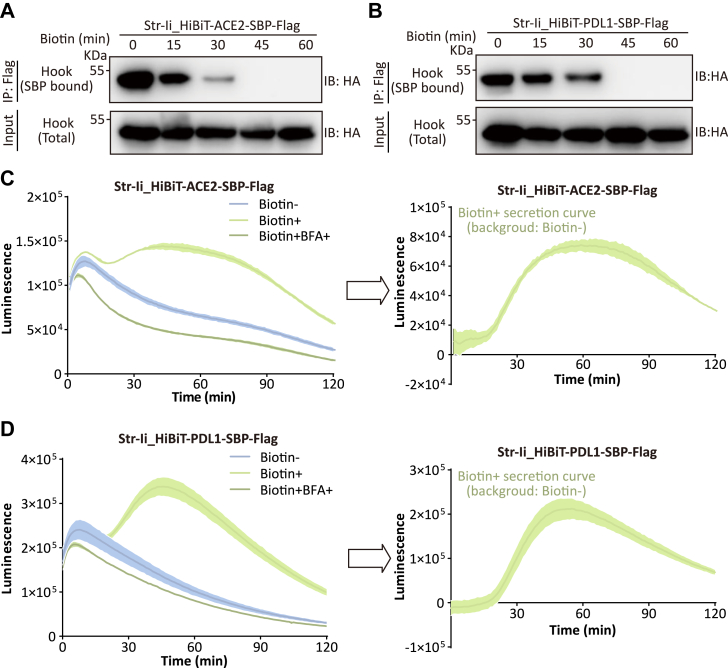
**Proof of concept of the strategy.***A* and *B*, immunoprecipitation with anti-FLAG of Str–Ii_HiBiT-ACE2-SBP-FLAG (*A*) or Str–Ii_HiBiT-PDL1-SBP-FLAG (*B*)-transfected 293T cells to capture HA-tagged Str–Ii hook bound by HiBiT reporter at indicated times after biotin addition, anti-HA antibody was used to detect the hook. *C* and *D*, Str–Ii_HiBiT-ACE2-SBP-FLAG (*C*) or Str–Ii_HiBiT-PDL1-SBP-FLAG (*D*)-transfected 293T cells were added by Nano-Glo HiBiT Extracellular Detection Reagent including the substrate furimazine and LgBiT as the manufacturer’s instruction, and luminescence was continuously monitored every minute over a 120-min period at 37 °C. Biotin-, treated without biotin; Biotin+, treated with 40 μM biotin; Biotin + BFA+, treated with 40 μM biotin and 20 μM brefeldin A. n = 3, shaded region around the curve represents SD. ACE2, angiotensin-converting enzyme 2; BFA, brefeldin A; PDL1, programmed death-ligand 1; SBP, streptavidin-binding peptide; Str, streptavidin.

To verify the feasibility of the constructs, the interactions of the hook and the reporters following biotin addition were assessed using coimmunoprecipitation (co-IP) assays. We added biotin and collected samples at 15-min intervals over the first 60 min to monitor the release of the HiBiT-tagged ACE2–PDL1 from the hook. Notable separation was observed at both 15- and 30-min time points, and the interactions became undetectable by 45 min for both (Fig. 2, *A* and *B*), indicating that our constructs effectively initiate the release of ACE2 and PDL1 from the ER. Building on this, we formally conducted the secretion dynamics experiments for ACE2 and PDL1 using a plate reader in the presence or the absence of the transport inhibitor, brefeldin A (BFA) (20). A no-biotin control group was included as the background, and secretion curves were plotted over a 120-min period (Fig. 2, *C* and *D*). As expected for this canonical secretory inhibitor, BFA—which redistributes cis/medial Golgi components to the ER within minutes (21)—completely abrogated secretion when coadministered with detection reagents. In contrast, pretreatment with 50 nM microtubule-depolymerizing agent, nocodazole (22) for 1 h significantly suppressed ACE2-HiBiT secretion (data not shown). This differential response—immediate blockade by BFA *versus* delayed suppression requiring nocodazole pretreatment—demonstrates the HiBiT-RUSH system's capacity to resolve mechanistically distinct secretory modulators, establishing its utility for evaluating diverse compounds targeting cellular secretory pathway. Notably, while our HiBiT-integrated RUSH strategy, like the classical RUSH system, is highly prone to reporter leakage, the introduction of a biotin-free control effectively provides a background subtraction that yields a clear secretion profile. In contrast, the classical RUSH system requires a more efficient retention mechanism to minimize fluorescence leakage and prevent it from confounding result interpretation (23).

### Identification of NPC1 as the lysosomal adaptor for ACE2 through HiBiT-integrated RUSH

**Figure 3:**
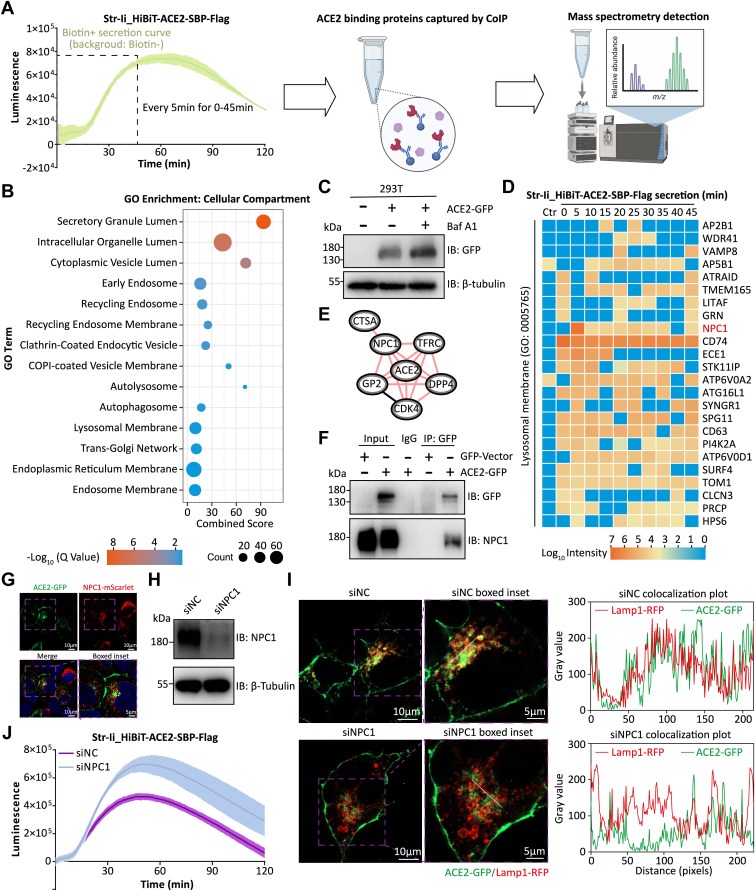
**Identification of NPC1 as the lysosomal adaptor for ACE2 through HiBiT-integrated RUSH.***A*, schematics of the screening strategy for ACE2 trafficking cofactors using Str–Ii_HiBiT-ACE2-SBP-FLAG-transfected 293T cells by coimmunoprecipitation/mass spectrometry (co-IP/MS). *B*, Gene Ontology (GO) enrichment analysis of secretion-related cellular compartment using the Enrichr platform (41). *C*, immunoblotting analysis of ACE2 levels in ACE2-GFP-transfected 293T treated with 200 nM bafilomycin A1 (Baf A1) for 6 h. Anti-GFP antibody was used to detect ACE2-GFP. *D*, heatmap illustrating the ACE2 trafficking cofactors at the lysosomal membrane (GO: 0005765). *E*, ACE2–NPC1 interaction network based on a previous study (27). *F*, immunoblotting analysis of ACE2 and NPC1 using co-IP in Huh7 cells. The indicated plasmids were transfected into Huh7 cells for 24 h, and NPC1 was pulled down by GFP-Vector or ACE2-GFP with GFP antibody. *G*, immunofluorescence analysis of ACE2 and NPC1 colocalization in Huh7 cells. ACE2-GFP and NPC1-mScarlet were cotransfected into Huh7 cells for 24 h and imaged using confocal microscopy. Colocalization plot profile was generated with ImageJ software. *H*, immunoblotting analysis of 293T cells transfected with scrambled siRNA (siNC) or NPC1 siRNA (siNPC1) for 48 h. *I*, Huh7 cells expressing ACE2-GFP and Lamp1-RFP were transfected with siNC or siNPC1 for 48 h, and the colocalization of ACE2 and Lamp1 was analyzed by confocal microscopy. Colocalization plot profiles were generated with ImageJ software. *J*, secretion dynamics assay to assess the function of NPC1 in ACE2 trafficking in Str–Ii_HiBiT-ACE2-SBP-FLAG-transfected 293T cells cotransfected with siNC or siNPC1. ACE2, angiotensin-converting enzyme 2; NPC1, Niemann–Pick complex 1; RUSH, Retention Using Selective Hooks; SBP, streptavidin-binding peptide; Str, streptavidin.

Next, we conducted co-IP/mass spectrometry (MS) at 5-min intervals over a 45-min period of secretion to define ACE2 interactomes along its trafficking route (Fig. 3*A*). As anticipated, Gene Ontology analysis revealed significant enrichment of cellular compartments associated with classic secretion, including the secretory granule lumen, cytoplasmic vesicle lumen, trans-Golgi network, and ER membrane. In addition, organelles related to the endosome, autophagosome, and lysosome were also enriched, likely reflecting the complexity of cellular trafficking processes (24) (Fig. 3*B*). The enrichment of autophagosomal and lysosomal cellular compartments particularly drew our attention, as these organelles directly mediate protein degradation and are supported by studies confirming that ACE2 undergoes both autophagic (25) and lysosomal degradation (26). Given that autophagic degradation typically occurs under cellular stress conditions, we excluded autophagosomes and focused specifically on lysosomal involvement. To directly demonstrate ACE2 undergoes lysosomal degradation, we treated ACE2–GFP-transfected 293T cells with 200 nM lysosomal acidification inhibitor, bafilomycin A1, for 6 h. This treatment effectively induced upregulation of ACE2–GFP protein levels, consistent with prior reports (25, 26) (Fig. 3*C*).

To identify the primary protein mediating ACE2–lysosome contact, we analyzed the interactome. Heatmap analysis revealed that over 20 lysosomal membrane proteins interact with ACE2 across various time points. Notably, proteins like CD74 interacted with ACE2 even at the 0-min time point (before biotin addition), likely because of reporter leakage as described previously. However, their interaction strength did not significantly increase upon biotin-induced ACE2 secretion, leading us to exclude them as key mediators. In contrast, NPC1 was the only protein that showed no binding to ACE2 at no-transfection or 0-min time point but exhibited a marked increase in binding at subsequent time points (Fig. 3*D*). In addition, prior studies investigating ACE2 interactors have suggested a direct ACE2–NPC1 interaction (27) (Fig. 3*E*). Co-IP (Fig. 3*F*) and immunofluorescence (Fig. 3*G*) analyses confirmed the interaction and binding between ACE2 and NPC1. As a 13-pass transmembrane lysosomal membrane protein, NPC1 was previously identified as a STING adaptor facilitating its lysosomal degradation, raising the possibility that NPC1 may similarly regulate ACE2 trafficking (28). To explore this further, we performed NPC1 knockdown using siRNA (Fig. 3*H*). This effectively disrupted ACE2–lysosome association (Fig. 3*I*), and subsequent trafficking assays revealed significantly enhanced ACE2 delivery to the PM in NPC1-deficient cells (Fig. 3*J*). Collectively, the HiBiT-integrated RUSH system enabled rapid and definitive identification of NPC1 as the lysosomal adaptor of ACE2.

### Cholesterol induces ACE2 lysosomal escape by downregulating NPC1

**Figure 4:**
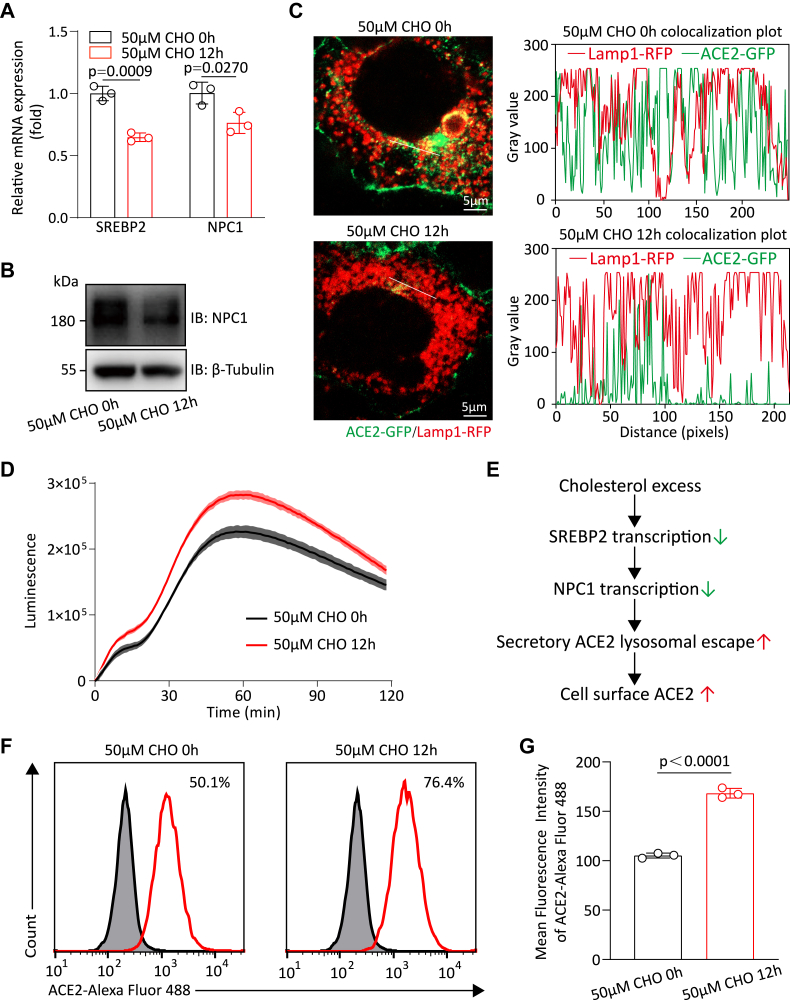
**Cholesterol (CHO) induces ACE2 lysosomal escape by downregulating NPC1.***A*, representative of three quantitative RT–PCR analysis of SREBP2 and NPC1 levels in Huh7 cells treated with 50 μM CHO for 12 h. Data are mean ± SD (n = 3 technical replicates). *B*, immunoblotting analysis of NPC1 levels in Huh7 cells treated with 50 μM CHO for 12 h. *C*, Huh7 cells expressing ACE2-GFP and Lamp1-RFP were treated with 50 μM CHO for 0 or 12 h, and the colocalization of ACE2 and Lamp1 was analyzed by confocal microscopy. Colocalization plot profiles were generated with ImageJ software. *D*, secretion dynamics assay to assess the ACE2 trafficking in Str–Ii_HiBiT-ACE2-SBP-FLAG-transfected 293T cells treated with 50 μM CHO for 12 h. *E*, diagram showing mechanisms of CHO-regulated ACE2 secretory pathway. *F*, representative of three flow cytometry analysis of cell surface ACE2 expression in Huh7 cells treated with 50 μM CHO for 12 h. *G*, mean fluorescence intensity (MFI) analysis of cell surface ACE2 expression. Data are mean ± SD (n = 3 independent biological replicates). Statistical significance was determined using an unpaired two-tailed Student’s *t* test. (∗*p* < 0.05, ∗∗*p* < 0.01, ∗∗∗*p* < 0.001, ∗∗∗∗*p* < 0.0001, and “ns” means not significant). ACE2, angiotensin-converting enzyme 2; NPC1, Niemann–Pick complex 1; SREBP2, sterol regulatory element–binding protein 2.

NPC1, a cholesterol transporter localized to the lysosome, primarily mediates the distribution of low-density lipoprotein–derived cholesterol (29, 30). Previous studies have shown that sterol regulatory element–binding protein 2 (SREBP2)—a master transcriptional regulator of cholesterol biosynthesis (31)—also controls NPC1 transcription (32, 33, 34). Consistent with this, elevated cholesterol levels suppress SREBP2 expression, leading to reduced NPC1 expression, as confirmed in our experiments (Fig. 4, *A* and *B*). This suggests that cholesterol-mediated NPC1 downregulation may induce ACE2 lysosomal escape, as revealed by immunofluorescence assay (Fig. 4*C*). Furthermore, the HiBiT-integrated RUSH assay revealed significantly enhanced ACE2 delivery to the PM in cholesterol-treated cells (Fig. 4*D*), defining a cholesterol-regulated secretory pathway *via* NPC1-dependent lysosomal sorting (Fig. 4*E*). In fact, a previous study has reported an upregulation of ACE2 in response to cholesterol increase (35), as reflected by our flow cytometry results using Huh7 cells with endogenous ACE2 expression (Fig. 4, *F* and *G*). Collectively, these results suggest that cholesterol induces ACE2 lysosomal escape through the downregulation of NPC1, which may contribute to increased viral entry of severe acute respiratory syndrome coronavirus 2, potentially through mechanisms beyond enhanced membrane fusion (36).

## Discussion

Since its introduction in 2012, RUSH has been widely used by researchers and has become a cornerstone method in the field of cellular secretion studies (10). However, its application still faces several limitations. One of the main challenges is achieving a perfect hook, as fluorescence leakage can compromise result interpretation (23). In addition, quantitative bioimaging remains a difficult task, especially since a single two-dimensional image cannot accurately reflect the three-dimensional nature of cells (37). Furthermore, cellular transport is a highly dynamic process, necessitating rapid and precise data acquisition. In this study, to investigate ACE2 secretion, we integrated HiBiT tag into the RUSH system, allowing the generation of quantitative secretion curves without the need for stable cell line construction or achieving a perfect anchor. This strategy is particularly effective for studying the transport dynamics of various cell membrane receptors and secretory proteins, as it can also be applied to the analysis of PDL1.

There are some technical notes for employing HiBiT to quantify the secretion of proteins of interest. First, the topology of the reporter must be carefully considered to ensure successful anchoring with the hook, while allowing the HiBiT tag to be exposed to the extracellular space for detection following its release. Second, the signal peptide of the target protein should not be overlooked, as it could lead to the removal of the HiBiT tag during protein processing. In addition, achieving a perfect hook is not strictly necessary, by including a biotin-free control as a background, accurate quantitative curves can be generated without the need to establish stable cell lines or utilize inducible expression systems.

Like most transmembrane receptors, the abundance of ACE2 on the cell membrane surface is influenced by multiple factors, including transcription, secretion, and degradation processes. During the coronavirus disease 2019 pandemic, researchers have conducted extensive investigations into ACE2. At the transcriptional level, Meng *et al.* (38) discovered that fibroblast growth factor 7 upregulates ACE2 transcription in pancreatic islets, whereas Brevini *et al.* (39) found that bile acids can enhance ACE2 transcription in cholangiocytes through farnesoid X receptor activation. At the degradation level, Jin *et al.* (25) identified that ACE2 SUMOylation modification protects it from autophagic degradation, and Dang *et al.* (40) showed that ubiquitin-specific protease 2 inhibits ACE2's proteasomal degradation. However, to date, no studies have elucidated the regulators of its secretion process. In this study, we developed a precise and efficient research method that simplifies the study of secretion processes. By accurately screening and validating the role of NPC1 in ACE2 secretion, we provide insights into the molecular mechanism by which high cholesterol upregulates ACE2 levels and offer new perspectives for the prevention and treatment of coronavirus disease 2019.

## Conclusions

In summary, the HiBiT-integrated RUSH system is characterized by its efficiency and accuracy, making it ideal for real-time monitoring of protein secretion in living cells. This method converts the cellular secretion process into a quantitative curve, providing an objective means for result interpretation. It is particularly effective for studying the transport dynamics of various cell membrane receptors and secretory proteins, facilitating the identification of both common and specific regulators of the cellular secretory process. Ongoing studies are focused on screening compound libraries using this strategy to identify potential interventions targeting the secretion of disease-associated proteins.

## Experimental procedures

### Plasmid construction

Str–Ii_HiBiT-ACE2-SBP-FLAG and Str–Ii_HiBiT-PDL1-SBP-FLAG were constructed by modification of the Str–Ii_SBP-EGFP-Golgin84 plasmid (addgene #65303). Specifically, the restriction enzymes AscI (G05F023; Gene-Protein Link) and XbaI (G05F265; Gene-Protein Link) were used to remove the existing reporter sequence, which was then replaced with the HiBiT reporter as designed previously. The vector and insert were subsequently ligated using the Seamless Cloning Kit (D7010; Beyotime), according to the manufacturer's protocol. In accordance with this method, ACE2-GFP was generated by inserting the ACE2 coding sequence into the pEGFP-N1 vector, and NPC1-mScarlet was constructed by subcloning the NPC1 gene into the pScarlet-N1 vector.

### Cell culture and transfection

293T cells (CRL-3216; American Type Culture Collection) and Huh7 cells (JCRB0403; Japanese Collection of Research Biosources Cell Bank) were cultured in Dulbecco’s modified Eagle’s medium (Eallbio) supplemented with 10% fetal bovine serum (ExCell Bio) and 1% penicillin–streptomycin solution (Eallbio). The authenticity of the cell lines was validated using short tandem repeat profiling. All cell lines were routinely tested for mycoplasma contamination to confirm the absence of contamination throughout the study. For transfection, the indicated plasmids and siRNAs were introduced into cells using jetPRIME transfection reagent (Polyplus; Illkirch-Graffenstaden) according to the manufacturer's protocol. The siRNA sequences used in this study were as follows:

siNC, UUCUCCGAACGUGUCACGUTT

siNPC1, ACCAAUUGUGAUAGCAAUATT.

### Coimmunoprecipitation/masss sectrometry

Cells were lysed in ice-cold NP-40 lysis buffer (P0013F; Beyotime) for 30 min on ice. The lysates were then clarified by centrifugation at 12,000*g* for 15 min at 4 °C. For IP, protein lysates were incubated with anti-FLAG M2 affinity gel (A2220; Merck Millipore) or GFP tag antibody (50430-2-AP, Lot: 00162326; Sanying) overnight at 4 °C with gentle rotation. The immune complexes were then washed three times with cold wash buffer to remove nonspecific binding. Then, the MS analysis was conducted by Shanghai Omicsolution Co, Ltd. Briefly, the elutes were digested with trypsin and analyzed by liquid chromatography–tandem mass spectrometer (Thermo Fisher Scientific). Peptide identification was performed using MaxQuant against the UniProt to identify the interacting proteins. Results were filtered for a false discovery rate of <1%. Comprehensive and detailed MS experimental information has been included in the data we deposited with the ProteomeXchange Consortium *via* its iProX partner repository, under the dataset identifier PXD058450. Identified proteins were further analyzed for Gene Ontology enrichment analysis using the Enrichr website (https://maayanlab.cloud/Enrichr/).

### Immunoblotting

The cells were lysed in radioimmunoprecipitation assay lysis buffer (P0013B; Beyotime) containing a protease and phosphatase inhibitor cocktail. The resulting lysates were separated by 8 to 12% SDS-PAGE, followed by transfer to polyvinylidene fluoride membranes. Membranes were blocked with 5% nonfat dry milk for 2 h at room temperature, then incubated with primary antibodies overnight at 4 °C. After washing, the membranes were probed with horseradish peroxidase–conjugated secondary antibodies (Cell Signaling Technology). Protein signals were detected using electrochemiluminescence reagent (Thermo Fisher Scientific) and visualized with a chemiluminescent imaging system (Tanon). Lysosome acidification inhibitor bafilomycin A1 (B10504; Psaitong) was used to demonstrate that ACE2 undergoes lysosomal degradation. The primary antibodies used in this study were as follows: HA tag antibody (51064-2-AP, Lot: 00101521; Sanying), NPC1 antibody (13926-1-AP, Lot: 00100574; Sanying), β-Tubulin (AC030, Lot: 9100030001; ABclonal). The specificity of all antibodies used in this immunoblotting was commercially validated by the manufacturers.

### Quantitative reverse transcription PCR analysis

Total RNA samples were isolated from cells with NucleoZOL (740404.200; MACHEREY-NAGEL). Then, 800 ng RNA was reverse transcribed using a TransScript All-in-One First-Strand cDNA Synthesis SuperMix for qPCR (AT341; TransGen Biotech), quantitative reverse transcription PCR was performed in triplicate using PerfectStart Green qPCR SuperMix (AQ601; TransGen Biotech). The RNA levels were normalized using ACTB as an internal control. The primers used in this study were as follows:

ACTB-Forward: 5′-TCTCCCAAGTCCACACAGG-3′,

ACTB-Reverse: 5′-GGCACGAAGGCTCATCA-3′;

NPC1-Forward: 5′-GCACCTTTTACCATCACTCCTG-3′,

NPC1-Reverse: 5′-GGCCACAGACAATAGAGCAGT-3′;

SREBP2-Forward: 5′-CTCCATTGACTCTGAGCCAGGA-3′,

SREBP2-Reverse: 5′-GAATCCGTGAGCGGTCTACCAT-3′.

### Secretion dynamics assay

293T cells were cultured in 10-cm culture dishes and transfected with 5 μg Str–Ii_HiBiT-ACE2-SBP-FLAG or 5 μg Str–Ii_HiBiT-PDL1-SBP-FLAG. After 24 h, cells were trypsinized and seeded into a 96-well white cell culture plate (FCP968; Beyotime) at an appropriate density, followed by overnight incubation for adhesion. For HiBiT signal detection, Nano-Glo HiBiT Extracellular Detection Reagent (N2421; Promega) was prepared by mixing Large BiT (1:200 dilution) and furimazine substrate (1:100 dilution) into Dulbecco’s modified Eagle’s medium containing 40 μM biotin (D8150; Solarbio). The medium was replaced, and the plate was immediately placed into a prewarmed plate reader (Tecan) at 37 °C. Luminescence was measured at 1-min intervals for 120 min. For experimental controls, 20 μM BFA (S1536; Beyotime) and 50 nM nocodazole (N80005; Psaitong) were added respectively to confirm that the signal is transport specific, and a no-biotin control group was included as the background signal.

### Immunofluorescence

ACE2-GFP (2 μg) and NPC1-mScarlet (2 μg) were cotransfected into Huh7 cells cultured in 35-mm confocal dishes. After 24 h, the cells were fixed with 4% paraformaldehyde (G1101; Servicebio), and nuclei were stained with Hoechst (C1025; Beyotime). Imaging was performed using the ZEISS LSM 980 confocal microscope. For ACE2-GFP and lysosome colocalization imaging, Lamp1-RFP (addgene #1817) was used to visualize lysosomes.

### Flow cytometry

For cell surface ACE2 analysis, Huh7 cells were washed with PBS twice and incubated with anti-ACE2 (21115-1-AP, Lot: 00087165; Sanying) antibody (1:100 dilution) at 4 °C for 30 min, followed by a secondary donkey anti-rabbit antibody (406416, Lot: B310664; BioLegend) conjugated to Alexa Fluor 488 (1:200 dilution) at 4 °C for 15 min. The cells were washed twice and analyzed on an Invitrogen Attune NxT Acoustic Focusing cytometer (Thermo Fisher Scientific). The specificity of all antibodies used in immunofluorescence staining was commercially validated by the manufacturers.

### Statistical analysis

All data are displayed as mean ± SD. Unpaired Student’s *t* tests were used for the statistical analysis between two groups using GraphPad software (version 8.4.2; GraphPad Software, Inc). Differences with *p* < 0.05 were considered to be statistically significant.

## Data availability

The data analyzed in the current study are available from the corresponding author upon reasonable request. The MS proteomics data for "Proteomic Analysis of ACE2-Interacting Proteins During ER-to-PM Trafficking" have been deposited with the ProteomeXchange Consortium *via* the iProX partner repository under dataset identifier PXD058450 (available at https://proteomecentral.proteomexchange.org).

## Supporting information

This article contains supporting information.

## Conflict of interest

The authors declare that they have no conflicts of interest with the contents of this article.

## Supporting information

Peptide data of mass spectral proteomics 1

Peptide data of mass spectral proteomics 2
